# Meeting report: GARNet/OpenPlant CRISPR-Cas workshop

**DOI:** 10.1186/s13007-016-0104-z

**Published:** 2016-01-27

**Authors:** Geraint Parry, Nicola Patron, Ruth Bastow, Colette Matthewman

**Affiliations:** GARNet, School of Biosciences, Cardiff University, Cardiff, UK; OpenPlant, John Innes Centre, Norwich Research Park, Norwich, UK; Synthetic Biology Laboratory, The Sainsbury Laboratory, John Innes Centre, Norwich Research Park, Norwich, UK

**Keywords:** CRISPR, Cas9, Gene Editing, Genetic Engineering

## Abstract

Targeted genome engineering has been described as a “game-changing technology” for fields as diverse as human genetics and plant biotechnology. One technique used for precise gene editing utilises the CRISPR-Cas system and is an effective method for genetic engineering in a wide variety of plants. However, many researchers remain unaware of both the technical challenges that emerge when using this technique or of its potential benefits. Therefore in September 2015, GARNet and OpenPlant organized a two-day workshop at the John Innes Centre that provided both background information and hands-on training for this important technology.

## Background

Over the past few years, genome engineering (GE), the process of making targeted modifications to the genome, its contexts or its outputs, has been described as a “game-changing technology*”* for fields as diverse as human genetics and plant biotechnology. The ability to introduce specific changes to genomic loci adds a level of precision not previously available to molecular biologists working in multicellular eukaryotes. Despite overwhelming scientific opinion that Genetically Modified (GM) plants are safe and provide environmental and socioeconomic benefits, they remain broadly unpopular outside of the scientific community [1–3]. This has been blamed both on inaccurate media reporting and public concerns over the ownership of technologies that underpin food production [4–6]. Given these political and public opinions, plant scientists are particularly hopeful about the future use of GE technologies, which are likely to enable precise genetic changes to be made without the ongoing requirement for foreign DNA to be integrated the genome.

However, despite some countries ruling that plants with targeted mutations may not be regulated as GM, there is still much uncertainty [7, 8]. Even as the technologies behind GE are being optimized, the scientific community is engaging with stakeholders to highlight potential positive uses, including how it might be used to develop better crops. This is exemplified by a policy statement from the UK’s Biotechnology and Biological Sciences Research Council (BBSRC) on “New Techniques for Genetic Crop Improvement” that outlines positive uses for GE technologies [9].

The experimental protocols needed to implement these powerful techniques are yet to be embraced by many plant science laboratories. To address this issue GARNet [10] and OpenPlant [11] collaborated to organise a workshop to explain the background of Clustered Regularly Interspaced Short Palindromic Repeat (CRISPR)/Cas technologies for GE in plants and to equip plant scientists with the skills required to implement Cas9-induced targeted mutagenesis. Over 140 researchers registered for the meeting, held at the John Innes Centre (UK), from as far-afield as Ireland and Poland, clearly demonstrating the appetite to apply these technologies to plant systems. The first day was open to all attendees and consisted of conventional ‘seminar-style’ presentations, while day two was a hands-on introduction for 30 researchers. This meeting was made possible by the kind support of *Plant Methods*.

## Day one presentations

The meeting was opened by Dr. Jim Haseloff from The University of Cambridge who introduced synthetic biology in plant systems and Dr. Nicola Patron of The Sainsbury Laboratory, Norwich (TSL), the primary organiser, who provided a historical perspective on GE technologies. The specifics of these technologies are discussed in detail as part of this *Plant Methods* thematic series. Keynote presentations were given by Professor Holger Puchta (Karlsruhe Institute of Technology) and Professor Bing Yang (Iowa State University) who each provided overviews and success stories from their own laboratories. These were followed by shorter talks from scientists at JIC, TSL and the University of Cambridge who are already working with CRISPR/Cas technologies.

Professor Puchta gave an inspiring talk that provided attendees with the history of his seminal work. He presented earlier work showing that induction of double strand breaks (DSBs) using site-specific endonucleases can enhance the freqeuncy of homologous recombination in plant cells through to his recent work using RNA-guided Cas9 nuclease to induce DSBs [12, 13]. He mentioned that the two most important molecular discoveries of his lifetime had been the Polymerase Chain Reaction (PCR) and GE technologies, the latter he described as having “hit him like a tsunami”. It was exciting to hear about his lab’s recent use of paired nickase variants of Cas9, which cut just one DNA strand, to induce larger endogenous deletions [13, 14]. Professor Puchta was extremely positive about the potential for GE and in his final perspectives noted that “Synthetic nuclease based DSB-induced DNA repair should be applicable for directed mutagenesis in all transformable plants”, and “in the long run synthetic nuclease-based GE will change plant breeding dramatically”. He also thought it possible that plants with targeted mutations might not be regulated in the same way as transgenic GM plants.

Professor Yang echoed this, presenting a letter from the United States Department of Agriculture (USDA) that informed him that the GE rice produced in his laboratory did not fall within its regulatory authority [14, 15]. Professor Yang documented his work on GE in maize and rice, showing that in cultivars where poor transformation efficiency was a significant bottleneck, GE technologies has sped up the process. He also described the induction of a large deletion of 245 kb in rice using RNA-guided Cas9 [15].

Dr. Laurence Tomlinson and Dr. Vladimir Nekrasov, both from TSL, presented their successful applications of RNA-guided Cas9 nuclease to induce targeted mutagenesis in tomatoes. Tomlinson’s work involved GA signaling whilst Nekrasov described the induction of targeted mutations to engineer pathogen-resistance. He took the audience through initial experimental design, through screening of putatively mutated plants to the identification of individuals showing resistance to powdery mildew. It took just 9 months to identify transgene-free, resistant plants with heritable mutations. Nekrasov confirmed that he and his supervisor, Professor Sophien Kamoun, are now investigating options to make their plants available to growers in regions where the pathogen is a significant problem, whilst also undertaking full-genome sequence analysis to determine if the plants contain any additional mutations. University of Cambridge PhD student, Bernando Pollak, introduced the liverwort *Marchantia polymorpha*, highlighting the ease by which its genome can be manipulated, as well as its potential as an easily engineerable chassis for synthetic biology. Many of the signaling pathways in *Marchantia* lack the redundancy seen in vascular land plants [16] and so it has huge potential as a tool for the study of plant signaling. Additionally, *Marchantia* is haploid for a large portion of its life cycle and thus the application of programmable nucleases such as RNA-guided Cas9 are even easier to apply. Dr.Oleg Raitskin (TSL) described experiments to further optimize RNA-guided Cas9 nuclease mediated mutagenesis in plants, including the assessment of orthologues and mutants of Cas9 that may expand the number of possible targets in the genome. He also introduced the concepts behind digital droplet PCR and its implementation in the rapid, quantitative assessment of mutations.

The final presentation was delivered by Edward Perello, Chief Business Officer of Desktop Genetics [17], a UK-based software company who develop tools to support the application of CRISPR-associated technologies. Mr. Perello announced that their guide RNA selection software, Guidebook, now supports six plant genomes (*Arabidopsis*, rice, maize, wheat, barley and *Physcomitrella*). Plant scientists were encouraged to use this software, which is free for academics, as well as to contact the Desktop Genetics team with feedback and requests for new features and genomes.

## Day two workshop

For the workshop on the second day, participants were given a detailed introduction to the methods used to induce targeted mutagenesis and gene deletions with RNA-guided Cas9 nuclease. This was a hands-on session designed to give the participants a full understanding of how to undertake three key aspects of the technique: selecting target sequences, constructing plasmid vectors, and screening target loci for induced mutations. The content was tailored for researchers working on any transformable plant species.

As well as discussing targeted mutagenesis, Dr. Patron provided an introduction to Type IIS mediated assembly methods for the facile construction of plasmid vectors. Dr. Patron is an advocate for the adoption of standards in bioengineering. She was the lead author on a recent manuscript that described a broadly agreed common genetic syntax for the exchange of DNA parts for plants [18]. In addition, Dr. Patron has contributed to a toolkit of standard parts for plants and created a series of informative online tutorials that introduces users to the Golden Gate Modular Cloning (MoClo) assembly standard [19, 20]. Participants were instructed in the use of published standard parts (Table 1), compatible with the MoClo binary plasmid backbones to build vectors for multiplexed Cas9-induced mutagenesis. The workshop materials have been provided on the GARNet website [21] but the main points are summarized below.

**Table 1:** Published standard parts for plants for use in the assembly of binary plasmid vectors for Cas9-mediated targeted mutagenesis

Name	Description	AddGene code	Publication
*Constitutive promoters (PROM) and strong 5′ untranslated regions (5UTR) for use in Cas9 and selectable markers gene cassettes*			
pICH51266	PROM (1.3 kb), 35s (Cauliflower Mosaic Virus) + 5UTR omega (Tobacco Mosaic Virus)	#50267	[20]
pICH51288	PROM (double), 35s (Cauliflower Mosaic Virus) + 5UTR, omega (Tobacco Mosaic Virus)	#50269	[20]
pICSL12009	PROM and 5UTR Ubiquitin (*Zea mays*)	#68257	[22]
pICH87633	PROM, nopoline syntase (*Agrobacterium tumefaciens*) + 5UTR, omega (Tobacco Mosaic Virus)	#50271	[20]
pICH85281	PROM + 5UTR, mannopine synthase (*A. tumefaciens*) + 5UTR, omega (Tobacco Mosaic Virus)	#50272	[20]
pICSL12006	PROM + 5UTR (Cassava Vein Mosaic Virus)	#50270	[20]
*Small RNA promoters for single guide RNAs*			
pICSL9003	PROM U6 (*Triticum aestivum*)	#68262	[22]
pICSL90002	PROM U6-26 (*Arabidopsis thaliana*)	#68261	[22]
*Coding sequences (CDS) of selectable markers genes*			
pICSL80037	CDS neomycin phosphotransferase II (*Escherichia coli*)	#68260	[22]
pICSL80036	CDS hygromycin phophotransferase II (*E. coli*)	#68259	[22]
piCH42222	Phosphinothricin acetyl transferase, (*Streptomyces hygroscopicus*)	#50328	[20]
pICH43844	CDS phosphinothricin acetyl transferase, (*S. hygroscopicus*) with intron from ACT2 (*A. thaliana*)	#50329	[20]
*3′ untranslated regions (3UTR) and polyadenylation signals (TERM) for use in Cas9 and selectable markers gene cassettes*			
pICH41414	3UTR, polyadenylation signal/terminator, 35s (Cauliflower Mosaic Virus)	#50337	[20]
pICH41421	3UTR + TERM, nopaline synthase (*A. tumefaciens*)	#50339	[20]
pICH41432	3UTR + TERM octopine synthase (*A. tumefaciens*)	#50343	[20]
piCH72400	3UTR + TERM g7 (*A. tumefaciens*)	#50338	[20]
*Cas9 coding sequence and single guide RNA scaffold*			
pICH41308 ::hCas9	CDS Cas9 (*Streptococcus pyogenes*)	#49770	[23]
pICSL11061	Single guide RNA scaffold	#46966	[24]
*Assembled selectable marker and Cas9 transcriptional units*			
pICSL11055	pICH51288 (double 35s) + pICH80037 (nptII) + pICH41421 (nos) *in* pICH47732 (Level 1 Position 1) backbone	#68252	[22]
pICSL11059	pICH51266 (35s) + PICSL80036 (hptII) + pICH41414 (35 s) *in* pICH47732 (Level 1 Position 1) backbone	#68263	[22]
pICSL11060	pICSL12006 (CsVMV) + pICH41308::hCas9 (Cas9) + pICH41414 (35s) *in* pICH47742 (Level 1 Position 2) backbone	#68264	[22]
pICSL11056	pICSL12009 (ZmUbi) + pICH41308::hCas9 (Cas9) + pICH41421(nos) *in* pICH47742 (Level 1 Position 2) backbone	#68258	[22]
pICH47742::2x35S-5′UTR-hCas9(STOP)-NOST	pICH51288 (double 35 s) + pICH41308::hCas9 (Cas9) + pICH41421(nos) *in* pICH47742 (Level 1 Position 2) backbone	#49771	[23]

## Designing single guide RNAs (sgRNAs) for use with *Streptococcus pyogenes* Cas9

1. The target sequence, which is integrated into the single guide RNA (sgRNA), consists of 20 nucleotides (nt). In the genome, target sequences must be located immediately 5′ of an ‘NGG’ sequence, known as the Protospacer Adjacent Motif (PAM) (Fig. 1). The 6–8nt immediately 5′ of the PAM are called the ‘seed region’ and should be 100 % identical to the target sequence. DSBs may still be induced at targets with one or more mismatches in the 5′ end of the target sequence. The induction of DSBs in sequences that do not exactly match the guide is known as ‘off-target activity’ and may be exploited for simultaneously inducing mutations in closely related sequences although the delivery of multiple sgRNAs that exactly match each target may be more successful.

**Fig. 1:**
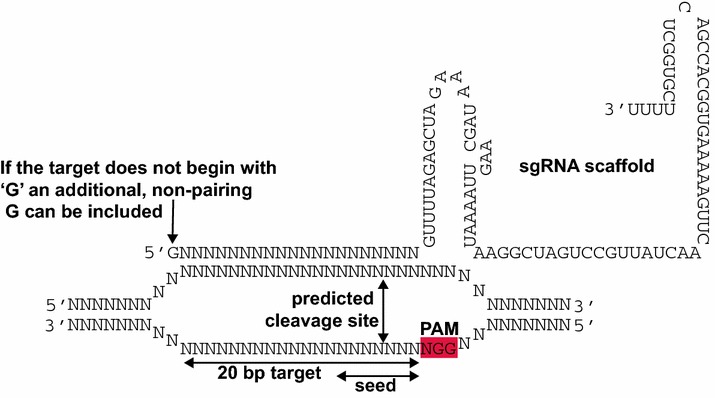
Interaction of a single guide RNA (sgRNA) expressed from a U6 promoter with its cognate genomic target (adapted from Belhaj et al. [23])
2. RNA polymerase III (RNAPol-III) dependent promoters are generally used to transcribe sgRNAs. This is because of their precise transcriptional start site. As the target sequence comprises the 5′ end of the sgRNA, the start of transcription must be preserved. For example, the transcriptional start site of the *Arabidopsis* U6-26 promoter is a ‘G’ and therefore the transcript will begin with a ‘G’. This nt does not necessarily need to pair with the genomic target. If the desired target sequence does not start with a ‘G’ an additional 5′ non-pairing ‘G’ can be included, extending the target to 21 nts (Fig. 1).
3. If specific sgRNA identification software is not available for the genome of interest, target sequences can be identified using many DNA analysis software packages by searching for the degenerate sequence ‘N(20)NGG’. Cas9 has been shown to preferentially bind sgRNAs containing purines in the last 4 nucleotides of the spacer sequence whereas pyrimidines are disfavoured [25]. Although unconfirmed in plant systems, users may wish to select targets rich in purines by searching for ‘N(12)R(8)NGG’.
4. For purposes of creating functional ‘knock-outs’, two or more sgRNAs can be designed to the same gene, thus creating a small deletion. Constructs with multiple sgRNAs, the Cas9 and selection genes as well as other transcriptional units can be easily assembled using the MoClo plasmid system and published standard parts (Table 1) [20, 22, 26].

Once the constructs have been assembled, they are delivered to plant cells using established protocols for the species of interest. Although transient transfection of plasmids and direct delivery of protein-RNA complexes to protoplasts have resulted in targeted mutagenesis [27, 28], regeneration from protoplasts has not yet been established for many plant species. The assembled genes may be integrated as a transgene raft. The resulting transformants can then be analysed for lesions at the target locus. The final part of the workshop was dedicated to simple, rapid techniques for the identification of induced mutations at target loci.

### Screening putatively mutagenised plants

1. Genomic DNA is purified and, if two sgRNAs were used, oligonucleoitide primers flanking the targets sites are used to PCR amplify the locus. Evidence of a deletion can be seen in the form of amplicons smaller than those obtained from a wild type control (Fig. 2a). The absence of the wildtype amplicon may indicate that the deletion was homozygous (Fig. 2a). The sequence of this band may confirm if both sister chromatids were repaired in the same way or if the plant is bialleic. If an amplicon corresponding to the wild-type is also present, the deletion may be heterozygous or, alternatively, the transgenes may be expressed in somatic tissues with cells in the sample showing multiple genotypes. In all cases the seeds will be collected and null-segregent progeny, which have not inherited the transgene, and (unless the deletion was homozygous in the primary transformants) progeny that have inherited the transgene analysed in the same way. The mutation can be classified as heritable and stable when progeny with the same mutant genotype as the parent are recovered and the transgene has been segregated out.

**Fig. 2:**
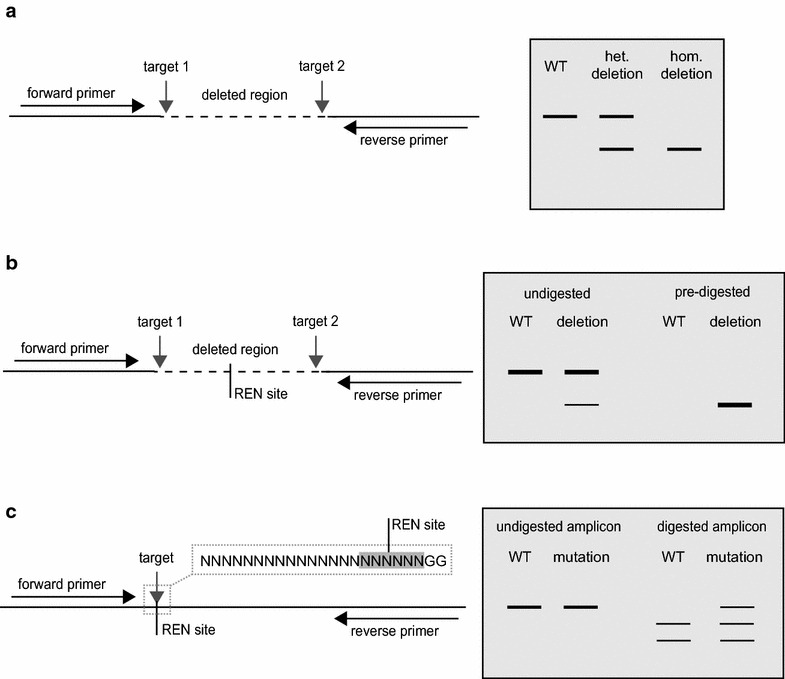
Detection of induced mutations**. a** If two single guide RNAs were delivered with the aim of deleting a fragment of DNA, oligonucleoitide primers flanking the targets can be used to PCR amplify the locus. Evidence of an amplicon, smaller that that obtained in a wildtype (WT) control is indicative of a deletion. The absence of an amplicon of equivalent size to the WT may indicate a homozygous deletion. **b** If the quantity of the deletion amplicon is low or absent, the genomic DNA can be digested with any restriction endonuclease (REN) with one or more recognition sites in the deletion region prior to PCR amplification. This will remove any wild-type sequence enabling the detection of deletions even if at low quantity in the sample. **c** Double strand breaks (DSBs) are most likely to occur three base pairs before the PAM in the seed-region of the target. Small insertion-deletion events at the target can be detected by digesting a PCR amplicon of the target locus with a REN for which the cognate sequence would be disrupted by imperfect repair of the DSB
2. Following PCR amplification, if there is no evidence of smaller band indicating a deletion then two experiments are possible: The first is to digest the purified genomic DNA with a restriction endonuclease with one or more recognition sites between the targets and to PCR amplify the locus with oligonucleotide primers designed to the flanking regions (Fig. 2b). This pre-digestion will remove any wild-type sequence enabling the detection of deletions from just a few cells in the sample. Such plants are highly likely to be chimeric and will need to be progressed to a second generation. The second method allows the detection of small insertion-deletion events at the target rather than a deletion. A DSB is most likely to occur three base pairs before the PAM in the seed-region of the target (Fig. 1). If there is a restriction endonuclease recognition site that would be disrupted by imperfect repair of the DSB, a PCR amplicon of the target locus can be digested with this enzyme. Any amplicon showing resistance to digestion with this enzyme can be sequenced (Fig. 2c). A researcher with sufficient foresight will try to design a target region that contains RE sites that could be used for subsequent screening. Again, the mutation can be classified as heritable and stable when progeny with the same mutant genotype as the parent are recovered and the transgene has been segregated out.

Mutations are detected in at least some cells of at least 5–20 % of primary transformants, with much higher frequencies reported for some species [29]. This rate is dependent on the effectiveness of the specific sgRNAs and species-specific factors including the level of expression of Cas9 and sgRNAs achieved in the tissue to which the transgene is delivered.

One of the main criticisms of programmable nucleases for the induction of targeted mutations is the potential for off-target activity. Although many plant species can be easily backcrossed to ‘clean up’ the genetic background as is done for chemical or radiation-induced mutagenesis, off-targets can only be identified by sequencing either related target sites or the whole genome. Nevertheless, there is little doubt that GE technologies offer immediate opportunities for increasing genetic diversity in crop plants and for understanding the function of plant genes. The take-away message from this workshop was that the technique has enormous potential, but that it can be technically challenging to implement. A post-workshop survey received many positive responses about the breadth of the talks and especially regarding the day two workshop. However, there are still knowledge gaps in the plant science community and therefore GARNet will be organising a further CRISPR-Cas workshop as part of its general meeting, to be held in September 2016 (http://www.GARNet2016.weebly.com).
